# Bid Promotes K63-Linked Polyubiquitination of Tumor Necrosis Factor Receptor Associated Factor 6 (TRAF6) and Sensitizes to Mutant *SOD1*-Induced Proinflammatory Signaling in Microglia^1^,^2^,^3^

**DOI:** 10.1523/ENEURO.0099-15.2016

**Published:** 2016-05-12

**Authors:** Sinéad Kinsella, Hans-Georg König, Jochen H.M. Prehn

**Affiliations:** Department of Physiology and Medical Physics, Centre for the Study of Neurological Disorders, Royal College of Surgeons in Ireland, 123 St. Stephen’s Green, Dublin 2, Ireland

**Keywords:** microglia, NF-κB, SOD1G93A protein, TLR, Toll-like receptor 4

## Abstract

Mutations in the *superoxide dismutase 1* (*SOD1*) gene contribute to motoneuron degeneration and are evident in 20% of familial amyotrophic lateral sclerosis cases. Mutant *SOD1* induces microglial activation through a stimulation of Toll-like receptors 2 and 4 (TLR2 and TLR4). In the present study, we identified the proapoptotic Bcl-2 family protein Bid as a positive regulator of mutant *SOD1*-induced TLR-nuclear factor-κB (NF-κB) signaling in microglia. *bid*-deficient primary mouse microglia showed reduced NF-κB signaling in response to TLR4 activation or exposure to conditioned medium derived from *SOD1* ^G93A^ expressing NSC-34 cells. Attenuation of NF-κB signaling in *bid-*deficient microglia was associated with lower levels of phosphorylated IKKα/β and p65, with a delayed degradation of IκBα and enhanced degradation of Peli1. Upstream of IKK, we found that Bid interacted with, and promoted, the K63-linked polyubiquitination of the E3 ubiquitin ligase tumor necrosis factor receptor associated factor 6 (TRAF6) in microglia. Our study suggests a key role for Bid in the regulation of TLR4-NF-κB proinflammatory signaling during mutant *SOD1*-induced disease pathology.

Bid promotes TLR4-NF-κB signaling by interacting with TRAF6 and promoting TRAF6 K63-linked polyubiquitination in microglia.

## Abstract (precis)

Mutations in the *superoxide dismutase 1* (*SOD1*) gene contribute to motoneuron degeneration and are evident in 20% of familial amyotrophic lateral sclerosis cases. Mutant *SOD1* induces microglial activation through a stimulation of Toll-like receptors 2 and 4 (TLR2 and TLR4).

## Visual Abstract

**Figure:**
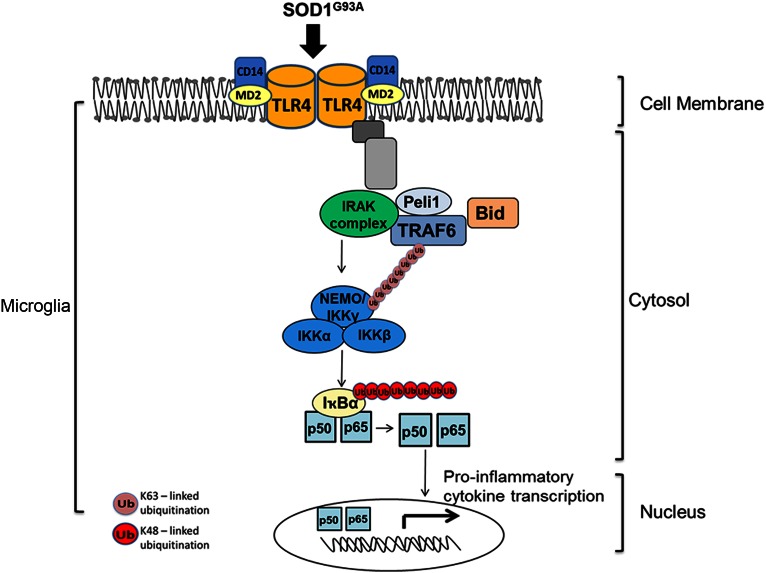

## Significance Statement

Recent work suggests that many proapoptotic proteins also function in other cell-signaling pathways and may affect inflammatory responses. Inflammation is a hallmark of Amyotrophic Lateral Sclerosis (ALS) disease models, and inflammation-associated markers in ALS patients are associated with more severe disease progression. Here we demonstrate that the proapoptotic Bcl-2 family protein Bid plays a crucial role in mutant *SOD1*-induced microglial activation, and delineate a novel signal transduction pathway activated by Bid involving K63-linked polyubiquitination of the E3 ubiquitin ligase tumor necrosis factor receptor associated factor 6 (TRAF6) and subsequent NF-κB activation.

## Introduction

Amyotrophic lateral sclerosis (ALS) is a fatal, progressive neurodegenerative disease, characterized by the selective death of upper motoneurons in the cerebral cortex and lower motoneurons in the brainstem and anterior horn of the spinal cord (Ferraiuolo et al., 2011). Mutations in the *superoxide dismutase 1* (*SOD1*) gene, linked to chromosome 21q22.1, account for ∼12–20% of the familial cases of ALS (Rosen et al., 1993; Renton et al., 2014). The cytotoxicity of mutant SOD1, a 153 aa cytosolic protein, is mainly described as a toxic gain-of-function mechanism (Tu et al., 1996). Mutations in *SOD1* lead to protein misfolding and aggregate formation (Rotunno and Bosco, 2013) and have been shown to induce endoplasmic reticulum (ER) stress and proteasomal dysfunction, which trigger several stress-activated disease processes in ALS (Kikuchi et al., 2006; Kieran et al., 2007). In addition, mutant SOD1^G93A^ has been shown to have marked misfolding effects on wild-type *SOD1* (Grad et al., 2011). Of note, it has become increasingly evident that non-cell autonomous toxicity mechanisms contribute to mutant SOD1-induced motoneuron degeneration (Liu et al., 2009; Grad et al., 2011). Activation of both astrocytes and microglia are implicated in ALS pathogenesis with glial cell crosstalk contributing to the burden of inflammation (Hensley et al., 2006; Evans et al., 2013; Brites and Vaz, 2014). Studies have identified microglia to be phenotypically neuroprotective at disease onset, however a proinflammatory activation state is soon acquired upon disease progression resulting in a chronic inflammatory pathology (Liao et al., 2012). Microglia expressing mutant SOD1 display elevated responses to inflammatory stimuli (Xiao et al., 2007; Sargsyan et al., 2009), and mutant SOD1^G93A^ secreted from motoneurons activates microglia and induces neurotoxicity (Zhao et al., 2010).

Accumulating evidence demonstrates that mutant SOD1 induces increased Toll-like receptor (TLR) expression in ALS (Liu et al., 2009; Casula et al., 2011). TLRs are the master regulators of the cellular innate immune response (Scheffel et al., 2012), and are key mediators of the initiation and propagation of the inflammatory cascade in response to bacterial, viral or microbial nucleic acids, known as pathogen-associated-molecular-patterns (Kielian, 2006). TLR activation initiates several cascades of intracellular pathways, one of which leads to NF-κB activation. NF-κB is a transcription factor and the major cytoplasmic facilitator of inflammatory stimuli. Recent evidence suggests that SOD1^G93A^ activates the transmembrane receptors TLR2 and TLR4 in a CD14-dependent manner (Zhao et al, 2010).

Previous studies observed increased levels of the Bcl-2 family member Bid (BH3-interacting domain death agonist) in the spinal cords of SOD1^G93A^ transgenic mice (Guégan et al., 2002; König et al., 2014). Bid is involved in the permeabilization of the mitochondrial outer membrane during death receptor activation, which leads to apoptosis (Wang et al., 1996). Recent research has suggested an immunoregulatory role of Bid (Mayo et al., 2011, Yeretssian et al., 2011; König et al., 2014). An interaction between Bid and the innate immune receptor nucleotide-binding and oligomerization domain was suggested, however this study did not demonstrate attenuated LPS-induced inflammation in the absence of Bid (Yeretssian et al., 2011). Reduced phagocytic functioning was revealed in *bid*-deficient microglia (Mayo et al., 2011), and Bid was shown to associate with the IKK complex upstream of NF-κB, specifically NEMO in intestinal epithelial cells (Yeretssian et al., 2011) and astrocytes (König et al., 2014). NEMO/IKKγ is the regulatory subunit of the IKK complex [IKKα, IKKβ, and IKKγ/NEMO (NF-κB essential modulator)], activation of which is central to NF-κB activation. We therefore set out to investigate the role of Bid as a positive regulator of mutant SOD1-induced TLR signaling in microglia, with a focus on Bid promotion of TLR4-NF-κB pathway activation.

## Materials and Methods

### Chemicals and antibodies

Common chemicals were obtained from Sigma-Aldrich unless otherwise stated. Antibodies used for Western blotting and immunohistochemistry include rabbit anti-TLR2 (Abcam, ab108998; 1:500), rabbit anti-TLR4 (Santa Cruz Biotechnology, sc-10741; 1:100), anti-CD11b (Abcam, ab8878; 1:400), anti-pIKKα/β (Cell Signaling Technology, 92465; 1:500), anti-IL-1β (Abcam, ab9722; 1:2000), anti-TNFα (Abcam, ab9635; 1:500), anti-Bid (Enzo Laboratories, AR-52; 1:1000), anti-Peli1 (Abcam, ab13812; 1:1000), anti-pp65 (Cell Signaling Technology, 30315; 1:500), anti-IκBα (Cell Signaling Technology, 9242; 1:500), anti-COX-II (BD Biosciences, 610203; 1:200), anti-TRAF6 (Santa Cruz Biotechnology, sc8409; 1:200), anti-α-tubulin (Sigma-Aldrich, T6199, 1:5000), anti-β-actin (Sigma-Aldrich, A3853; 1:5000), anti-GAPDH (Abcam, ab8245-100;1:5000).

### Cell culture

BV-2 cells, a murine cell line alternative to primary microglia commonly used to model the neuroinflammatory role of microglia (Henn et al., 2009), were cultured in RPMI containing 1% penicillin-streptomycin, 2 mm l-glutamine, and 10% fetal bovine serum. NSC-34 cells were kindly donated from the Shaw laboratory at the University of Sheffield, UK (originally generated by Cashman et al., 1992), and were cultured in DMEM with 4.5 g/L glucose, 1% penicillin-streptomycin, 2 mm l-glutamine, and 10% fetal bovine serum. HEK293 deficient in TLR4 expression and HEK293/hTLR4-MD2-CD14 cells (kindly donated by Andrew Bowie, Trinity College Dublin, Ireland) were cultured in RPMI containing 1% penicillin-streptomycin, 2 mm l-glutamine, and 10% fetal bovine serum. HEK293-TLR4 media was supplemented with Blasticidin (InvivoGen; 10 µg/ml), Normocin (InvivoGen; 100 µg/ml), and Hygromycin B Gold (InvivoGen; 50 µg/ml).

### Mixed glial-cell culture and microglial isolation

All procedures involving animals were conducted under a license from the Department of Health and Children, as well as the Health Products Regulatory authority in Ireland. Procedures were reviewed by the Ethics Committee of the Royal College of Surgeons in Ireland. Mixed glial cultures were prepared from the cortices of P0–P2 WT and *bid^−/−^* mice of mixed sexes on a *C57BL/6* background. *bid^−/−^* mice were generated in the laboratory of Dr Andreas Strasser, WEHI, Melbourne, Australia (Kaufmann et al., 2007). The cortices were dissected and the meninges were removed before incubation in Trypsin-EDTA at 37°C for 10 min. DMEM-F12/l-glutamine (Gibco, Life Technologies) containing penicillin-streptomycin (1%; Sigma-Aldrich) and fetal bovine serum (10%; Sigma-Aldrich) was added to the cortices before trituration and passage through a 40 µm nylon cell strainer (BD Falcon). The cells were centrifuged at 300× *g* for 6 min and the pellet was resuspended in DMEM-F12 containing Pen/Strep and FBS. The cells were plated at a density of ∼4 cortices/T75 flask, and treated with M-CSF (10 ng/ml; R&D Systems), and GM-CSF (20 ng/ml; R&D Systems) from 1 day post-dissection to promote microglial proliferation (Suzumura et al., 1990). Microglia were isolated using the shaking method. The media containing the detached and floating microglia was collected and centrifuged at 800 × *g* for 6 min. The pelleted isolated microglia were resuspended in DMEM-F12 (containing FBS and Pen/Strep) and plated at a density of 3.5 × 10^5^ cells/well in a 6-well plate. The remaining cells were passaged and cultured in the absence of M-CSF and GM-CSF to obtain astrocyte cultures.

### Mixed primary motoneuron preparation

Mixed primary cultures enriched for motoneurons were prepared from *C57BL/6* murine WT E12 embryos by the dissection of the ventral horn of the spinal cord and subsequent purification, as previously described (Sebastià et al., 2009). In brief, the ventral horns were incubated with 0.025% trypsin in Neurobasal media for 10 min, followed by gentle dissociation in the presence of 0.1 mg/ml DNase1. The dissociated motoneurons were centrifuged at 300 × *g* for 3 min and resuspended in complete Neurobasal media supplemented with 2 mm GlutaMAX, 2% horse serum, 2% B27, GDNF (Promega, Catalog #2781; 2 ng/ml), CNTF (R&D Systems, Catalog #557-NT-10; 1 ng/ml), 100 U/ml penicillin and 100 μg/ml streptomycin. The cells were seeded at a density of 0.25 × 10^6^ cells/well in a 24-well plate pre-coated with polyornithine and laminin and cultured at 37° C in 5% CO_2_. Motoneurons were harvested for Western blotting at 7 DIV.

### Generation of SOD1^G93A^ conditioned media

NSC-34 cells were reverse transfected with 5 μg/well CFP or SOD1^G93A^-CFP plasmid using Lipofectamine 2000 (1:2, DNA–Lipofectamine; Invitrogen) in Opti-MEM (Invitrogen) at a density of 4 × 10^5^ cells/well in a 6-well plate. After 4 h of incubation with the transfection mix, the cells were washed once with serum containing DMEM and incubated overnight in fresh DMEM containing full serum. The cells were then washed with serum free DMEM and incubated in the latter for 3 d before conditioned media was collected and centrifuged at 800 × *g* for 5 min to remove cellular debris. Conditioned media was stored at –20°C or concentrated on 10 kDa size exclusion columns (10K, Amicon Ultra, UFC501096, Millipore) centrifuged at 4°C at 14,000 rpm for 45 min, boiled in Laemmli buffer for 5 min, and ran on an acrylamide gel for quantification of SOD1 content in the media.

### Overexpression of SOD1^G93A^ in microglia

BV-2 cells were transfected using electroporation (Amaxa Nucleofector II, Lonza). Briefly, the cells were harvested and a density of 5 × 10^6^ cells/5 μg plasmid was used for each cuvette. The cells were washed with RPMI (centrifuged at 90 × *g* for 10 min) and the pellet was resuspended in 100 μl RPMI including 5 μg pcDNA3-CFP (13030; Addgene), pcDNA-SOD1wt-CFP, or pcDNA-SOD1^G93A^-CFP and transfected via electroporation (program “A-023”). The transfected cells were carefully triturated in 1 ml of RPMI and placed in the incubator for 1 min before being plated on 6-well plates. Cells were incubated for 8 h and lysed using Buffer RLT (Qiagen RNA easy kit) for mRNA extraction, or at 24 h in RIPA buffer for protein analysis. Primary microglia were transfected with CFP or SOD1^G93A^-CFP (0.35 × 10^6^ cells/5 µg plasmid) using Lipofectamine (2 µl/µg plasmid) and OptiMEM. The cells were incubated with the transfection mix for 2.5 h before being replaced with serum containing media. The cells were lysed in RLT buffer 24 h post-transfection for RNA extraction and qPCR analysis.

### qPCR analysis

RNA was extracted using Qiagen RNAeasy kit and cDNA was synthesized using random primers and Superscript RTII (Invitrogen). qPCR analysis was carried out on a Roche Lightcycler 2.0 using SYBRgreen (Quantitect SYBRgreen kit, Qiagen). Two microliters of each cDNA sample and 18 µl of the appropriate Mastermix [1 µl 10 µm primer (forward and reverse), 10 µl SYBRgreen PCR Mix, 7 µl RNase-free H_2_0] was added to give a total volume of 20 µl per capillary tube. *gapdh* was used as an internal control for each sample analyzed. The cycle parameters were 95°C for 15 min, 94°C for 15 s, 57°C for 25 s, 72°C for 30 s, and the annealing temperature for each primer was 57°C. The primers used were as follows: *gapdh (mouse)* forward 5′ AACTTTGGCATTGTGGAAGG 3′, reverse 5′ ACACATTGGGGGTAGGAACA 3′; *tlr2 (mouse)* forward 5′ GCGGACTGTTTCCTTCTGAC 3′, reverse 5′ CCAAAGAGCTCGTAGCATCC 3′; *tlr4 (mouse)* forward 5′ GCATGGCTTACACCACCTCT 3′, reverse 5′ GTCTCCACAGCCACCAGATT 3′. Primers were designed using Primer3 (http://biotools.umassmed.edu/bioapps/primer3_www.cgi), and are between 150 and 250 base pairs, optimized for SYBR detection.

### Immunohistochemistry

Primary microglia were fixed with 3% paraformaldehyde for 12 min at 37°C, washed three times for 5 min in PBS, and blocked for 30 min at room temperature in blocking solution [5% horse serum, 0.3% Triton X-100 (Sigma-Aldrich) in PBS]. After three washes with PBS the primary antibody [anti-CD11b (1:200) or anti-pIKKα/β (1:500)] was diluted in PBS 1% horse serum, 0.3% Triton X-100 in PBS and added to the wells (150 µl/well of 24-well plate), before incubation for 2 h at room temperature. The cells were washed three times with PBS and incubated in the dark with an AlexaFluor secondary antibody solution for 1 h at room temperature (1:500) in 1% horse serum, 0.3% Triton X-100 in PBS (anti-mouse AlexaFluor 488, Invitrogen A10037), anti-rabbit AlexaFluor 488 (Invitrogen, A21441), anti-rat AlexaFluor488 (Invitrogen, A11006), anti-mouse AlexaFluor 568 (Invitrogen, A10037), anti-rabbit AlexaFluor 568 (Invitrogen). Hoechst was used as a nuclear stain (Hoechst 33342, Invitrogen). Mean fluorescence was analyzed using ImageJ (NIH; imagej.nih.gov/ij).

### Co-immunoprecipitation and pull-down experiments

Overexpression of tumor necrosis factor receptor associated factor 6 (TRAF6) was performed by transfection of the pCMV5-FLAG-wt-TRAF6 vector in BV-2 cells. In short, BV-2 cells were transfected with FLAG-wt-TRAF6 (Addgene, 21624) or pCMV5-FLAG (1.5 µg/3 × 10^5^ cells) using Lipofectamine. The cells were stimulated with LPS for 1 or 4 h (1 µg/ml BV-2, or 100 ng/ml primary glia and astrocytes). Cells were lysed in RIPA buffer (Tris 50 mm, NaCl 150 mm, SDS 0.1%, sodium-deoxycholate 0.5%, Triton X-100 or NP-40 1%, plus 1:100 Protease Inhibitor, Sigma-Aldrich). Co-immunoprecipitation or pull down experiments were performed using DynaBeads Protein G [35 μl of Dynabeads/sample (100–400 μg protein), Life Technologies, 10007D], and a magnetic rack (Life Technologies). The beads were washed in RIPA buffer and incubated with 5 μg primary antibody (in 200 μl PBS for 30 min at room temperature). The beads were washed three times for 5 min in RIPA buffer and equal amounts of protein (100 μg) were incubated rotating for 2 h at room temperature (in a total volume of 750 μl). The protein was eluted from the beads and denatured in RIPA buffer plus 1× Laemmli buffer by incubating for 10 min at 70°C. The tubes were placed on the magnetic rack and the denatured supernatant was collected for gel electrophoresis.

### siRNA transfection

An siRNA targeting Bid, sequence ACACGACUGUCAACUUUAU, was designed using an algorithm optimized for siRNA selection (Reynolds et al., 2004), and obtained from Sigma-Aldrich. Briefly, BV-2 cells were transfected with 100 µM siRNA/3 × 10^5^ cells using Lipofectamine. Optimal silencing of *bid* was determined by qPCR analysis to be 48 h post-transfection. An siRNA consisting of a scrambled nucleotide sequence was used as the control in siRNA experiments.

### Western blot

Media was aspirated from the cells and the wells were washed gently with PBS and lysed with 80 µl/well of 6-well plate RIPA buffer for Western blot analysis. The cell lysates were incubated on ice for 15 min and centrifuged at 4°C at 13,000 rpm. The supernatants were used to determine the protein concentration by BCA assay (Micro BCA protein determination kit, Thermo Scientific). Laemmli buffer was added to each sample and the samples were boiled for 5 min and loaded onto 10%, 12%, or 15% polyacrylamide gels as appropriate. Semi-dry transfer was performed on to PVDF membrane for 1.5 h at 18 V. Membranes were exposed to Ponceau S and blocked in 3% milk for 1 h. Primary antibodies in 3% milk were incubated either overnight at 4°C or 2 h at room temperature. The membranes were washed in TBS-Tween 20 (0.1%), and placed in 3% blocking solution containing the appropriate secondary antibody (peroxidase-conjugated anti-mouse IgG, anti-rabbit IgG, or anti-goat IgG, Sigma-Aldrich; 1:5000, as appropriate) for 2 h at room temperature. The membrane was washed three times for 5 min in TBS-Tween 20, exposed to ECL Chemiluminescent Reagent (Millipore) for 5 min and imaged on a LAS-3000 Imager (Fuji). Quantification of protein levels were calculated from optical density measurements from Western blot experiments and normalized to respective loading control (α-Tubulin, β-actin, or GAPDH).

### Dual-luciferase assay

BV-2 cells were cotransfected by reverse transfection with renilla and firefly luciferase reporter gene vectors (renilla luciferase plasmid: firefly luciferase plasmid, 1:12) in Opti-MEM (Sigma-Aldrich; 1 µg plasmid per well/100 μl) using X-tremeGENE HP Reagent (Roche; 2 µl/µg plasmid). Following 5 h incubation with transfection mix the cells were washed and incubated with BV-2 growth media (RPMI + 10% FBS, 1% l-glutamine and 1% Pen/Strep) overnight. BV-2 cells were treated with 100 µm Bid Inhibitor (BI-6C9, Sigma-Aldrich), resuspended in DMSO, at a concentration 100 μm 30 min prior to treatment with LPS (1 µg/ml; Sigma-Aldrich, Catalog #L4391). BV-2 cells were lysed in 1× Passive Lysis Buffer (Promega) and luminescence was measured using Dual-Luciferase Assay Kit (Promega). HEK293 and HEK294-TLR4 cells were similarly cotransfected with constitutive renilla (phRL-TK) and firefly κB-luciferase reporter gene vectors [1:12 in OptiMEM using Lipofectamine (2 µl/1 µg plasmid)] by reverse transfection for 3 h before the cells were allowed to recover overnight in full serum media. After being washed gently with serum-free media both HEK293 and HEK293-TLR4 cells were stimulated with CFP or SOD1^G93A^-CFP conditioned media for 8 or 24 h before being lysed in passive lysis buffer, with luminescence measured using the method as above.

### Proximity ligation assay

The *in situ* proximity ligation assay (PLA) was carried out using the Duolink system (DUO92101-1KT, Sigma-Aldrich). This technique allows the identification of proximity between epitopes of proteins in a complex *in vitro* by using hybridization between oligonucleotides linked to secondary antibodies when bound to primary antibodies against two specific proteins in close proximity ≤40 nm *in situ* (Soderberg et al., 2006). Here we used mouse anti-TRAF6 (1:100) and rabbit anti-Bid (1:500; Abcam, ab62469) as primary antibodies to determine close proximity. BV-2 cells were plated in a 96-well plate and transfected with either pCMV-Myc-FLAG or pCMV-FLAG-TRAF6*wt* (Plasmid 21624, Addgene) at a concentration of 0.075 μg/2 × 10^4^ cells. Twenty-four hours post-transfection the cells were exposed to serum free media 2 h prior to treatment with either vehicle (1× PBS) or LPS (1 μg/ml). The cells were immunostained as described above. Controls included combinations of mouse anti-TRAF6 (1:100) plus rabbit anti-HA-tag (1:500; Santa Cruz Biotechnology, sc805), and rabbit anti-Bid plus mouse anti-IRF2 (1:500; Santa CruzBiotechnology, sc101069). PLA probes were diluted in primary antibody diluent (1% horse serum and 0.3% Triton x-100 in PBS) at a ratio of 1:10 and incubated for 1 h at 37°C. The ligation stock was diluted (1:5) with high-purity H_2_O and ligase 1:40. Twenty microliters per well of ligation–ligase solution was incubated with the cells at 37°C for 30 min. The cells were incubated with the amplification solution (1:5 with high-purity H_2_O and 1:40 polymerase) for 100 min at 37°C. The cells were washed three times with PBS and Hoechst was used as a nuclear stain (1:1000 in PBS, Hoechst 33342). The wells were imaged in order to detect amplification dots representing TRAF6-Bid close-proximities and analysis was performed using ImageJ software.

### Statistical analysis

Statistical analysis was performed using GraphPad Prism software or MATLAB software (v2014b, MathWorks) as applicable. Results are represented as mean ± SEM. Statistical significance was determined using the tests as detailed in the respective figure legends and Table 1 (*p ≤ 0.05).

**Table 1.** Statistical table

Label	Data structure	Type of test	Significance
Fig. 1A	Nonparametric	Paired two-tailed *t* test	*n* = 8–10 cultures*p* = 0.0156
Fig. 1B	Nonparametric	Paired two-tailed *t* test	*n* = 8–9 cultures*p*= 0.007
Fig. 1E	Nonparametric	Paired two-tailed *t* test	*n* = 6 cultures*p*= 0.031
Fig. 1F	Nonparametric	Paired two-tailed *t* test	*n* = 6 cultures*p*= 0.031
Fig. 1H	Nonparametric	Paired two-tailed *t* test	*n* = 3 cultures*p*= 0.0106
Fig. 1J	Parametric	One-way ANOVA, Tukey *post-hoc* test	*n* = 3 culturesp= 0.0021
Fig. 1K	Parametric	Paired two-tailed *t* test	*n* = 11 wells from 2 separate experiments*p*= 0.0216 HEKTLR4 cells CFP vs SOD1G93A
Fig. 2C	Parametric	One-way ANOVA, Bonferroni’s multiple-comparison test	*n* = 4–6 cultures*p*= 0.027
Fig. 3C	Parametric	Three-way ANOVA, Tukey *post hoc* test	*n* = 3 cultures*p*= 0.0147 WT vs *bid*
Fig. 3G	Parametric	One-way ANOVA, Tukey’s multiple-comparison test	*n* = 7 cultures*p*= 0.0162
Fig. 3H	NonparametricGrubbs outlier removal	Kruskal–Wallis test, Dunn’s multiple-comparison *post hoc* test	*n* = 6–16 culturesDMSO LPS vs DMSO Veh*p* < 0.0001BI Veh vs DMSO Veh*p* = 0.6926BI LPS vs DMSO Veh*p* = 0.5933BI Veh vs DMSO LPS*p* < 0.0001BI LPS vs DMSO LPS*p*= 0.0162BI LPS vs BI Veh*p* = 0.0162
Fig. 4*B*	Parametric	Three-way ANOVA, Tukey *post hoc* test	*n* = 3 cultures*p* = 0.000823WT vs *bid*
Fig. 5*F*	Nonparametric	Kruskal–Wallis, Dunn’s multiple-comparison *post hoc* test	*p* = 0.0378minus LPS vs plus LPS
Fig. 6*F*	Nonparametric	Kruskal–Wallis, Dunn’s multiple-comparison *post hoc* test	*n* = 3 cultures*p* = 0.0509

## Results

### SOD1^G93A^ overexpression increases TLR2 and TLR4 levels and COX-2 activation in microglia

**Figure 1.**
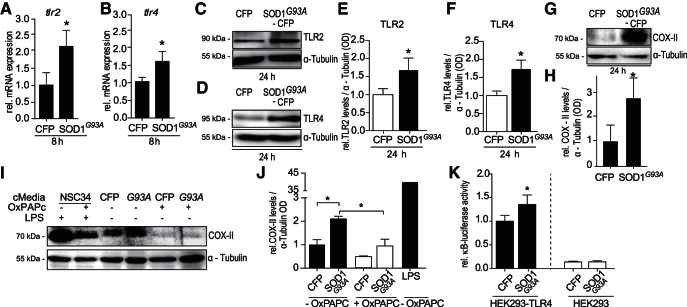
TLR2 and TLR4 expression is increased in response to SOD1^G93A^ overexpression. BV-2 cells were transiently transfected with CFP or SOD1^G93A^-CFP plasmids and harvested at 8 or 24 h post-transfection. ***A***, *tlr2* mRNA expression in transfected BV-2 cells was measured at 8 h post-transfection by qPCR analysis, relative to control gene *gapdh* (*n* = 8–10 wells pooled from 3 separate experiments; Kruskal–Wallis, Dunn’s multiple-comparison *post hoc* test). ***B***, *tlr4* mRNA was analyzed by qPCR analysis of BV-2 lysates 8 h post-transfection with CFP or SOD1^G93A^-CFP (*n* = 8–9 wells pooled from 2 separate experiments). Samples were normalized to internal control *gapdh.* ***C*, *E***, TLR2 protein levels in CFP or SOD1^G93A^-CFP-transfected BV-2 cells analyzed by Western blot. BV-2 cells were lysed in RIPA buffer 24 h post-transfection via nucleofection (normalized to α-Tubulin OD, *n* = 6 wells pooled from 4 separate experiments; *p* = 0.031, two-tailed paired *t* test). ***D***, ***F***, TLR4 protein levels in CFP or SOD1^G93A^-CFP-transfected BV-2 cells analyzed by Western blot. BV-2 cells were lysed in RIPA buffer 24 h post-transfection via nucleofection (normalized to α-Tubulin OD n = 6 wells pooled from 4 separate experiments; *p* = 0.031, two-tailed paired *t* test). ***G***, ***H***, COX-II levels were assessed in SOD1^G93A^ overexpressing BV-2 cells (*n* = 3 cultures from 2 separate platings; *p* = 0.016, two-tailed paired *t* test). BV-2 cells were transfected with CFP or SOD1^G93A^-CFP via electroporation and lysed in RIPA buffer 24 h post-transfection and prepared for Western blot analysis. ***I***, ***J***, COX-II levels in TLR2- and TLR4 -inhibited BV-2 cells following stimulation with CFP or SOD1^G93A^ cMedia. BV-2 cells were treated with OxPAPC (30 µg/ml) simultaneous to cMedia treatment. Cells were lysed 24 h post-cMedia stimulation and prepared for Western blot analysis (*n* = 3 wells, *p* = 0.002, one-way ANOVA, Tukey’s multiple comparison *post hoc* test). ***K***, NF-κB activity in TLR4-deficient HEK293 and HEK293-TLR4-stably expressing cells stimulated with SOD1^G93A^ conditioned media. HEK293 and HEK293-TLR4 were cotransfected with κB-RE-luciferase and RLTK-Renilla-luciferase for normalization for 24 h and subsequently stimulated with CFP or SOD1^G93A^ cMedia for 8 or 24 h. The cells were lysed in passive lysis buffer and measured as κB-dependent firefly activity normalized to renilla luciferase activity per well (*n* = 11 wells pooled from 2 separate experiments; *p* = 0.021, two-tailed paired *t* test between HEK-TLR4 treated cells). Conditioned media was generated by overexpression of CFP or SOD1^G93A^-CFP vectors in NSC-34 cells, with serum-free conditioned media collected 3 d post-transfection.

To explore the role of TLRs in SOD1 disease pathology, we analyzed *tlr2* and *tlr4* expression in response to overexpressed SOD1^G93A^ in microglia. BV-2 cells transiently transfected with mutant SOD1^G93A^ exhibited a significant increase in *tlr2* (Fig. 1*A*; 2.17-fold ± 0.84) and *tlr4* (Fig.1*B*; 1.55-fold ± 0.37) mRNA levels. We also observed increased TLR2 (Fig. 1*C*,*E*; 1.6-fold ± 0.51) and TLR4 (Fig. 1*D*,*F*; 1.72-fold ± 0.37) protein levels in SOD1^G93A^-transfected BV-2 cells compared with CFP-control transfected cells, analyzed by Western blotting. These results suggested that the presence of SOD1^G93A^ primed microglia for increased TLR2 and 4 signaling.

Cyclooxygenase-II (COX-II), an enzyme that is induced in response to proinflammatory stimuli (Nadjar et al., 2005; Laflamme et al., 1999), is recognised as a target gene of NF-κB activation (Nakao et al., 2000; Nadjar et al., 2005). We found significantly increased COX-II levels in response to transient transfection of BV-2 cells with SOD1^G93A^ (Fig. 1*G*,*H*; 2.07-fold ± 1.11 increase), and following paracrine stimulation with SOD1^G93A^ conditioned media (Fig. 1*I,J*; 2.1-fold ± 0.33 increase). COX-II levels were not significantly induced in TLR2 and TLR4-inhibited BV-2 cells, using the small molecule inhibitor OxPAPC, which were stimulated with NSC-34-derived SOD1^G93A^-conditioned media (Fig. 1*I*,*J*). OxPAPC (1-palmitoyl-2-arachidonyl-sn-glycero-3-phosphorylcholine), an oxidized phospholipid, was used here to selectively inhibit TLR2 and TLR4 dimerization by interacting with MD2, LPS-binding protein, and CD14, an extracellular receptor which is indispensable for both TLR2 and TLR4 dimerization (Fitzgerald et al., 2004; Erridge et al., 2008), thus blocking TLR2- and TLR4-induced activation. Additionally, COX-II levels were significantly lower in OxPAPC treated cells stimulated with SOD1^G93A^-conditioned media compared with control cells exposed to the same stimulus (Fig. 1*I*,*J*; 2.2-fold ± 0.407 decrease), suggesting that SOD1^G93A^ elicited its toxicity through TLR-NF-κB signaling. Next HEK293 cells that are deficient in TLR4 (Stack and Bowie, 2012) and HEK293 cells stably expressing TLR4 were exploited to demonstrate the requirement of TLR4 in SOD1^G93A^-mediated NF-κB activation. HEK293 cells stably expressing TLR4 showed increased NF-κB activation, as measured by NF-κB reporter firefly luciferase readout in response to stimulation in paracrine with SOD1^G93A^ (Fig. 1*K*; 1.35-fold ± 0.31 increase), with no increased NF-κB activation observed in HEK293 cells devoid of TLR4. These data support the previous findings by Zhao et al. (2010) and points toward a TLR4-dependent mechanism of action of SOD1^G93A^.

### *bid^−/−^* microglia show reduced NF-κB activation upon TLR4 stimulation

**Figure 2.**
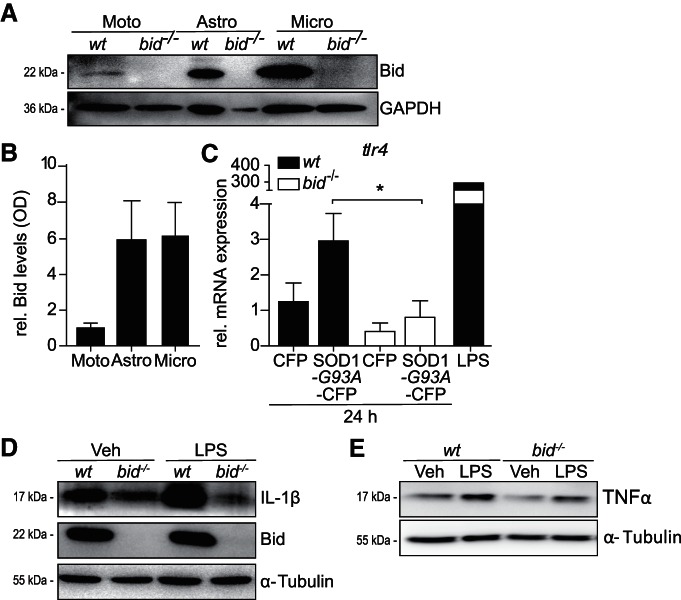
Bid is highly expressed in microglia, SOD1^G93A^-overexpressing *bid*-deficient microglia express reduced *tlr4* mRNA levels compared with wild-type, and levels of proinflammatory IL-1β and TNFα are reduced in LPS-stimulated *bid^−/−^* microglia. ***A***, Representative Western blot showing Bid levels and (***B***) quantification of optical density analysis of Bid levels in motoneurons, astrocytes and microglia. Primary mixed motoneurons were harvested at 7DIV, and purified astrocytes and isolated microglia were lysed 2 d post-plating to allow time for the cells to become quiescent. Bid protein levels were examined, by Western blot, in purified astrocytes and microglia at rest compared with motoneurons. Bid protein levels were assessed by measuring the optical densities of each sample relative to that of the respective loading control (***B***; GAPDH or β-actin, experiment was repeated 3 times with similar results). ***C***, *tlr4* mRNA expression in wild-type and *bid^−/−^* primary microglia overexpressing CFP or SOD1^G93A^-CFP, analyzed by qPCR. Samples were normalized to internal control *gapdh* (*n* = 4–6 wells pooled from 2 separate experiments; *p* = 0.027, one-way ANOVA, Bonferroni’s multiple-comparison *post hoc* test). ***D***, IL-1β levels were analyzed by Western blot in wild-type and *bid^−/−^* microglia 4 h post-LPS treatment. ***E***, TNFα levels were analyzed in wild-type and *bid*-deficient microglia 4 h post-LPS stimulation. Wild-type and *bid^−/−^* microglia were stimulated with vehicle, LPS (100 ng/ml) for 4 h before being harvested with RIPA lysis buffer and used for Western blot analysis.

To investigate the involvement of Bid in microglial immune responses we examined Bid protein levels in resting WT mixed motoneurons, astrocytes, and microglia. We found higher levels of Bid protein in astrocytes and microglia (Fig. 2*A*,*B*; astrocytes 5.9-fold ± 2.78, microglia 6.2-fold ± 2.08), verifying previous findings (König et al., 2014). To investigate the effects of Bid deficiency on SOD1^G93A^-induced microglial *tlr4* mRNA expression we generated WT and *bid^−/−^* primary microglia cultures and transiently transfected the microglia with CFP or SOD1^G93A^-CFP. *tlr4* expression was higher in SOD1^G93A^-overexpressing wild-type microglia compared with *bid-*deficient microglia (Fig. 2*C*; 2.9-fold ± 1.2 increase vs 0.8-fold ± 0.9 decrease). *tlr4* mRNA levels did not significantly increase in wild-type microglia overexpressing SOD1^G93A^ compared with CFP-transfected microglia, but increased TLR4 protein levels were detected in SOD1^G93A^-overexpressing BV-2 cells (Fig. 1*D*,*F*).

To additionally investigate the effect of a lack of Bid on TLR4-induced proinflammatory signaling in microglia both WT and *bid^−/−^* microglia were stimulated with the well characterized TLR4 agonist LPS (Takeuchi et al., 1999). Of note, both TLR2 and TLR4 signal to NF-κB via the MyD88-dependent pathway (Akira and Hoshino, 2003). Wild-type and *bid^−/−^* microglia were stimulated with LPS for 4 h and lysed for Western blot analysis of the cytokine IL-1β and TNFα levels. IL-1β was used as a proinflammatory marker as IL-1β has been shown to be rapidly secreted in response to LPS (Dinarello, 1997) and to be critical in the NF-κB induction of COX-II-derived prostaglandins in the CNS (Laflamme et al., 1999). TNFα is a proinflammatory cytokine secreted in response to LPS (Sawada et al., 1989), and microglial secretion of TNFα is shown to induce motoneuron death *in vitro* (He et al., 2002). Analysis revealed substantially reduced proinflammatory cytokine IL-1β and TNFα production (Fig. 2D,E) in *bid*-deficient microglia compared with wild-type cells 4 h post stimulation with LPS.

**Figure 3.**
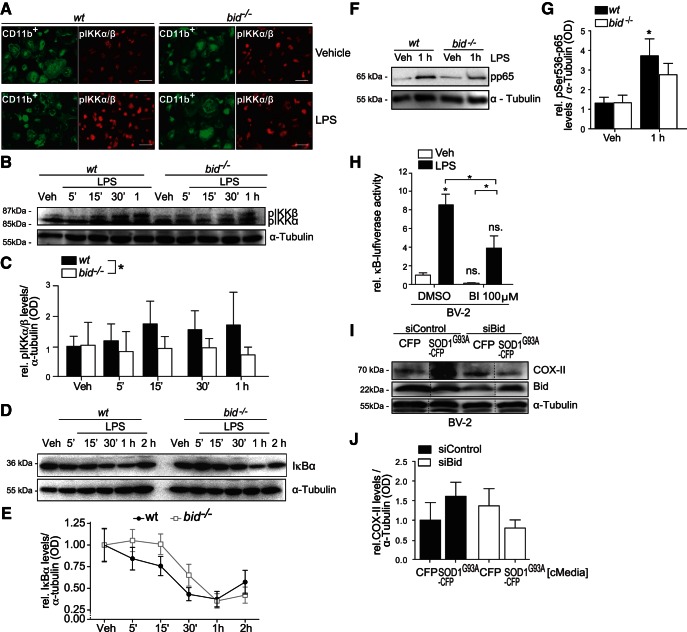
Reduced phosphorylation of IKKα/β, p65 and delayed IκBα degradation and reduced NF-κB activation in *bid^−/−^* microglia. ***A***, Primary wild-type and *bid^−/−^* microglia were stimulated with LPS for 5–30 min in serum-free media before being fixed in 3% paraformaldehyde and stained with anti-phosphorylated IKKα/β (pIKKα/β) and anti-CD11b. Immunohistochemistry analysis of anti-pIKKα/β mean fluorescence on CD11b-positive cells is depicted. Scale bar, 50 µm. ***B***, ***C***, Primary wild-type and *bid^−/−^* microglia were treated with LPS for 5, 15, 30 min, or 1 h in serum-free media before being lysed for Western blot analysis of pIKKα/β protein levels (*n* = 3 pooled from 3 separate experiments; *p* = 0.014, 3-way ANOVA, Tukey *post hoc* test). ***D***, ***E***, Wild-type and *bid^−/−^* microglia were stimulated with LPS for 5 min to 2 h in serum-free media, lysed in RIPA buffer and IκBα levels were analyzed by Western blot (*n* = 3–4 wells from 3–4 separate experiments). ***F***, ***G***, Wild-type and *bid^−/−^* microglia were stimulated with LPS for 1 h before being lysed in RIPA buffer. pp65 levels were assessed by Western blot (*n* = 7 wells pooled from 6 separate experiments; *p* = 0.0162, one-way ANOVA, Tukey’s *post hoc* test). ***H***, BV-2 cells were cotransfected with NF-κB-RE-luciferase and renilla-luciferase plasmids for 24 h and subsequently treated with LPS for 24 h. The cells were lysed in passive lysis buffer and NF-κB activation was quantified by dual luciferase assay (represented as relative κB-dependent firefly activity, *n* = 6–16 wells pooled from 2 separate experiments, 2 outliers removed, Grubbs test followed by Kruskal–Wallis and Dunn’s multiple-comparison *post hoc* test). ***I***, ***J***, COX-II levels in Bid-depleted BV-2 cells stimulated with CFP or SOD1^G93A^ cMedia. BV-2 cells were transfected with an siRNA targeting Bid (“siBid”) or a scrambled control siRNA (“siControl”), and stimulated with cMedia 48 h post-siRNA transfection, when Bid levels were optimally reduced. Twenty-four hours post-cMedia treatment the cells were lysed in RIPA and COX-II levels were measured. Dashed line indicated irrelevant lanes spliced out. Quantification of optical density was normalized to anti-α-tubulin for each Western blot (*n* = 3, from 3 separate experiments).

Because microglia are the first line of immune response in CNS pathogenesis we investigated the effect of *bid*-deficiency on microglial TLR4-NF-κB activation following acute LPS stimulation. LPS-induced activation of the IKK complex leads to IκBα degradation, in a phosphorylation-induced and proteasome-dependent manner (Alkalay et al., 1995; Brown et al., 1995; Yang et al., 2003). Wild-type and *bid^−/−^* microglia were stimulated with LPS for 5 min, fixed in 3% formaldehyde and stained with anti-CD11b and anti-pIKKα/β. CD11b is a cluster of differentiation molecule that is highly expressed in cells of the innate immune system, including macrophages and microglia, and is involved in mediating inflammation by regulating migration and adhesion properties. CD11b was used as a microglial marker and the fluorescence intensity of pIKKα/β was measured on CD11b-expressing cells. Overall decreased immunofluorescence of phosphorylated IKKα/β was noted in LPS-stimulated Bid-deficient microglia compared to wild-type CD11b-positive cells (Fig. 3*A*). Western blot analysis of microglia stimulated acutely with LPS, for 5, 15, 30, min or 1 h, showed significantly less phosphorylation of IKKα/β and less phosphorylated p65 in *bid^−/−^* microglia compared with WT upon LPS stimulation from 5 min to 1 h (Fig. 3*B*,*C*). The degradation profile of the NF-κB inhibitor IκBα was measured as an indicator of TLR4-NF-κB activation (Fig. 3*D*,*E*) and demonstrated that *bid*-deficiency delayed signal-induced degradation of IκBα noted at 30 min and 1 h post LPS stimulation, confirming previous results (Mayo et al., 2011). Interestingly there was a delay in recovery of IκBα levels at 2 h post LPS stimulation in the *bid^−/−^* microglia compared with wild-type, suggesting a delay in the positive feedback response in the absence of *bid*. Additionally, the phosphorylation of p65 was significantly induced in wild-type microglia but not in *bid*-deficient microglia 1 h post LPS stimulation (Fig. 3*F*,*G*; 3.7-fold ± 1.3 increase vs 2.7-fold ± 0.8 increase).

NF-κB transactivation potential, as measured by κB-response element-dependent luciferase expression, was also significantly reduced when Bid was inhibited using 100 μm of the small molecule Bid inhibitor (BI-6C9) prior to LPS stimulation (24 h) in BV-2 cells (Fig. 3*H*; BI-6C9 + LPS decreased 3.00 ± 1.858-fold compared with DMSO + LPS). COX-II levels were assessed in Bid-depleted BV-2 cells following stimulation in paracrine with SOD1^G93A^. BV-2 cells were transfected with siRNA targeting Bid followed by stimulation with SOD1^G93A^ in cMedia. Western blot analysis determined reduced COX-II levels in SOD1^G93A^-treated BV-2 cells when Bid was depleted compared with control siRNA-transfected BV-2 cells exposed to the same SOD1^G93A^ stimulus (Fig. 3*I*,*J*; 1.6-fold ± 0.8 increase vs 0.8-fold ± 0.6 decrease).

### Reduced levels of Peli1 in *bid^−/−^* microglia upon TLR4 activation

**Figure 4.**
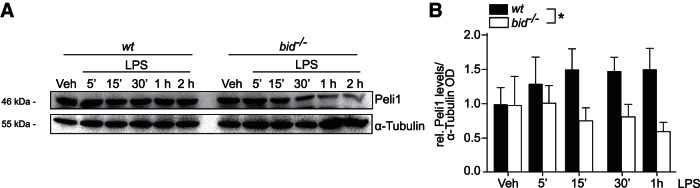
Peli1 levels are reduced in *bid^−/−^* microglia upon LPS stimulation. ***A***, ***B***, Peli1 levels were analyzed by Western blot analysis in wild-type and *bid^−/−^* microglia upon acute LPS stimulation (***B***; *n* = 3 cultures from 3 separate platings; three-way ANOVA, Tukey’s multiple-comparison *post hoc* test). Cells were stimulated with LPS (100 ng/ml) in serum-free media for the relevant treatment time points, lysed in RIPA buffer and prepared for Western blot analysis.

A number of E3 ubiquitin ligases regulate NF-κB signaling and activation. Peli1 is responsible for catalyzing the K63-linked polyubiquitination of the Interleukin-1 receptor-associated kinase (IRAK) complex (Moynagh, 2009) and has been described as a key regulator of CNS inflammatory pathogenesis (Song and Qian, 2013). Wild-type and *bid^−/−^* microglia were stimulated with LPS acutely as above, and lysed for Western blot analysis. Overall, Peli1 levels were significantly lower in *bid-*deficient microglia at early time-points post LPS stimulation (from 5 min to 1 h post-treatment) compared with *wild-type* microglia (Fig. 4*A*,*B*; WT microglia 1.49 ± 0.57-fold increased vs 0.59 ±0.46-fold decreased in *bid^−/−^* at 1 h LPS time point).

### Bid promotes TRAF6-mediated polyubiquitination

**Figure 5.**
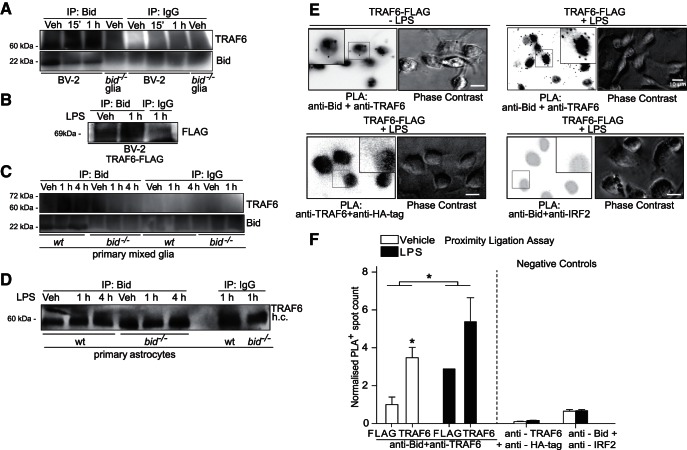
Bid associates with TRAF6 in microglia and astrocytes, as shown by coimmunoprecipitation and PLA. ***A***, Coimmunoprecipitation of Bid and TRAF6 in BV-2 cells. BV-2 cells were stimulated with LPS (1 µg/ml) for 15 min or 1 h and Bid was immunoprecipitated. Negative controls included anti-Bid immunoprecipitation from *bid-*deficient mixed glia lysates, and IgG immunoprecipitation from all samples. Cells were lysed in RIPA buffer and analyzed for TRAF6 content after immunoprecipitation of Bid. ***B***, Coimmunoprecipitation of Bid and TRAF6 in BV-2 cells overexpressing TRAF6-FLAG. BV-2 cells were stimulated with LPS (1 µg/ml) and lysed in RIPA buffer. FLAG was detected by Western blotting and represents TRAF6FLAG immunoprecipitated with Bid in BV-2 cells. An IgG immunoprecipitation was included as a negative control. ***C***, Coimmunoprecipitation of Bid and TRAF6 in WT and *bid-*deficient primary mixed glia stimulated with LPS for 1 and 4 h (100 ng/ml). Samples were lysed post-LPS stimulation and Bid was immunoprecipitated from the lysates. The samples were analyzed for TRAF6 content by Western blotting. IgG immunoprecipitation was carried out as an additional negative control. ***D***, Coimmunoprecipitation of Bid and TRAF6 in wild-type and *bid^−/−^* astrocytes. Purified astrocytes were stimulated with LPS (100 ng/ml) for 1 and 4 h, and lysed in RIPA buffer for Bid immunoprecipitation. The samples were analyzed for TRAF6 by Western blotting. ***E***, Representative images of PLA and phase contrast in TRAF6-FLAG overexpressing BV-2 cells immunostained with anti-Bid and anti-TRAF6 (*n* = 2 wells/condition, 4 fields of view per well). Negative control representative images of PLA in TRAF6-FLAG overexpressing BV-2 cells immunostained with anti-TRAF6 and anti-HA-tag (*n* = 1 well/condition, 6 fields of view-LPS, 1 field of view + LPS), or immunostained with anti-Bid and anti-IRF2 (*n* = 1 well/condition, 7 fields of view). Scale bar, 10 µm. ***F***, Quantification of PLA interactions in BV-2 cells. BV-2 cells were transfected with TRAF6-FLAG or empty FLAG vector and stimulated with LPS for 1 h. The cells were fixed with 3% paraformaldehyde, incubated with anti-Bid and anti-TRAF6 and PLA was quantified (significant increase of PLA dots in TRAF6 transfected versus control transfected cells and vehicle vs LPS treated cells, two-way ANOVA). Negative controls included immunostaining with anti-Bid plus anti-IRF2, and anti-TRAF6 plus anti-HA-tag.

Previous studies have identified Bid and IKKγ/NEMO association in response to inflammatory stimuli in intestinal epithelia (Yeretssian et al., 2011), as well as astrocytes. Here we identified an interaction with an upstream mediator of NEMO activation, TRAF6 (Goh et al., 2012). TRAF6, a member of the TRAF family of intracellular signaling adaptor proteins, is critical for K63-linked ubiquitination of both Peli1 and the IRAK complex (Conze et al., 2008; Goh et al., 2012; Moynagh, 2014). We found that Bid and TRAF6 coimmunoprecipitated in BV-2 cells and WT glia (Fig. 5*A*–*C*). This interaction between TRAF6 and Bid was additionally investigated in astrocytes by coimmunoprecipitation. Here we observed that TRAF6 immunoprecipitated with Bid in wild-type astrocytes upon LPS stimulation for 1 h, an interaction that was not seen in *bid-*deficient astrocytes (Fig. 5*D*). Additionally, we used proximity-ligation assays to explore the association of Bid with TRAF6 proteins within microglia *in situ*. Here, a significant increase in Bid and TRAF6 close-proximities was detected upon LPS stimulation in BV-2 cells (Fig. 5*E*,*F*; 1.9-fold increase ± 1.24).

**Figure 6.**
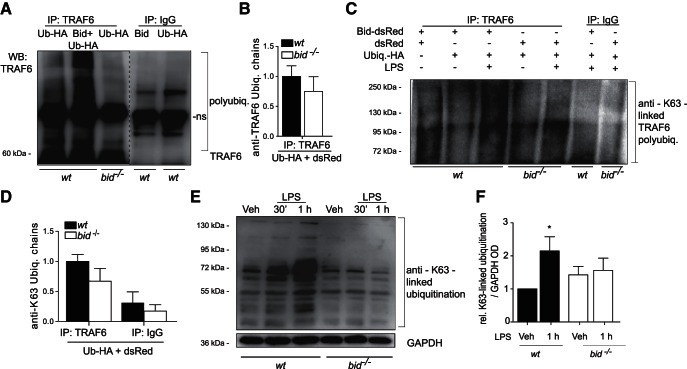
Bid promotes K63-linked polyubiquitination of TRAF6. ***A***, TRAF6 polyubiquitination levels in wild-type and *bid^−/−^* glia. TRAF6 was immunoprecipitated and TRAF6 polyubiquitination was assessed in WT mixed glia overexpressing Bid-dsRed plus Ubiquitin-HA (HA-Ub), and wild-type glia overexpressing ubiquitin-HA plus dsRed. *bid^−/−^* glia were transfected with ubiquitin-HA plus dsRed. Immunoprecipitation of IgG was used as an immunoprecipitation control. Single membrane cut as represented by dashed line. ***B***, Quantification of TRAF6-linked ubiquitin chains, as measured by OD in wild-type and *bid^−/−^* glia transfected with ubiquitin-HA plus dsRed (*n* = 2 pooled from 2 separate experiments). ***C***, Wild-type and *bid^−/−^* glia were transfected with the following plasmids, Bid plus dsRed, ubiquitin-HA plus dsRed or Bid plus ubiquitin-HA plasmids for 24 h and subsequently stimulated with LPS for 1 h. The cells were lysed in RIPA buffer. TRAF6 was immunoprecipitated from the transfected cell lysates and the samples were analyzed by Western blot. The membrane was exposed to an anti-K63-linked ubiquitin-specific antibody, which identified the K63-linked ubiquitination of TRAF6. ***D***, Quantification of TRAF6 K63-linked ubiquitin chains, as measured by optical density in wild-type and *bid^−/−^* glia transfected with ubiquitin-HA plus dsRed (*n* = 2–3 from 2–3 separate experiments). ***E***, Representative Western blot of total K63-linked ubiquitination levels in WT and *bid-*deficient microglia upon LPS stimulation. ***F***, Quantification of OD analyses of total K63-linked polyubiquitination in wild-type and *bid^−/−^* microglia following LPS stimulation (*n* = 3 cultures from 3 separate experiments; *p* = 0.050, one-way ANOVA, Dunn’s multiple-comparison *post hoc* test). Wild-type and *bid^−/−^* microglia were treated with LPS (100 ng/ml) for 30 min or 1 h before lysis in RIPA buffer and K63-linked ubiquitination levels were analyzed probing with anti-K63-linked ubiquitin-specific antibodies in Western blotting experiments.

Ubiquitination is a post-translational modification that involves the formation of ubiquitin linkage chains and leads to a variety of biological processes depending on which of the seven lysine residues of ubiquitin (K6, K11, K27, K29, K33, K48, K63) are covalently conjugated to form a polyubiquitin chain (Ikeda and Dikic, 2008). In addition, recent studies have identified a hybrid K63/linear (K63/M1)-linked ubiquitin chain formation in IKK complex signaling and subsequent NF-κB activation (Emmerich et al., 2013). As the polyubiquitination of TRAF6 is essential for the positive regulation of downstream proteins of the TLR-NF-κB pathway, such as NEMO, TAK1, and Peli1 we next assessed the levels of TRAF6 polyubiquitination in wild-type glial cells overexpressing Bid (Fig. 6*A*). Higher levels of TRAF6 polyubiquitination were present in wild-type glia compared with *bid-*deficient cells (Fig. 6*A*,*B*; 0.75-fold ± 0.41 decrease). Overexpression of Bid promoted TRAF6 polyubiquitination in glial cells, however, basal levels of Bid also showed increased TRAF6 polyubiquitination compared with *bid*-deficient glia (Fig. 6*B*,*D*). In addition, the impact of the absence of Bid on TRAF6 K63-linked autoubiquitination was investigated. Wild-type and *bid^−/−^* glial cells overexpressing Bid and/or Ubiquitin-HA were stimulated with LPS for 1 h and TRAF6 was immunoprecipitated from these samples. Subsequent Western blot analysis of immunoprecipitated samples revealed an increased K63-linked polyubiquitination of TRAF6 in LPS stimulated wild-type microglia overexpressing ubiquitin-HA compared with *bid^−/−^* glia overexpressing ubiquitin-HA (Fig. 6*C*,*D*; 0.67-fold ± 0.3 decrease). Next, the levels of total K63-linked ubiquitin chains were assessed in wild-type and *bid*-deficient microglia upon acute stimulations with LPS for 30 min and 1 h (Fig. 6*E*,*F*; 2.1-fold ± 0.42 increase vs 1.5-fold ± 0.36 increase 1 h post-LPS stimulation). The microglia were lysed and analyzed by Western blot, using an anti-K63-linked ubiquitin chain specific antibody. Lower levels of total K63-linked ubiquitin chains were observed when Bid was absent in microglia.

## Discussion

In this study, we demonstrate that Bid positively mediates microglial TLR4-NF-κB signaling, highlighting a nonapoptotic role of Bid in regulating immune responses in the CNS. Bid promotes the K63-linked polyubiquitination of TRAF6 upstream of IKK and absence of Bid attenuates the TLR4-induced NF-κB proinflammatory microglial response.

There is an emerging emphasis on the role of the innate immunity in driving neurodegenerative disease progression (Nguyen et al., 2002; Zhao et al., 2010), with mounting evidence implicating a central role of microglia in ALS pathogenesis (Beers et al., 2006; Di Giorgio et al., 2007; Glass et al., 2010). TLR2 and TLR4 mediate ALS-linked mutant SOD1 toxicity, as extracellular mutant SOD1^G93A^ activates microglia in a CD14-dependent manner (Zhao et al., 2010; Lee et al., 2015). Here we show increased TLR2 and TLR4 levels following overexpression of SOD1^G93A^ in BV-2 cells. Moreover, the data presented demonstrates increased COX-II levels in response to SOD1^G93A^, elicited through both autocrine and paracrine stimulation, further consolidating the previous findings presenting TLR2, TLR4, and NF-κB activation in mutant SOD1 treated microglia (Frakes et al., 2014; Lee et al., 2015). A lack of induction of Bid in microglia upon mutant SOD1 or LPS stimulation was observed (data not shown), indicating that the constitutively high levels of microglial Bid are sufficient for the signaling cascade to respond to TLR4-induced stimulation.

Recent studies proposed that Bid positively regulates the immunological profile of macrophages and epithelial cells, and Bid-deficiency resulted in decreased proinflammatory cytokine mRNA levels in response to LPS in microglia (Mayo et al., 2011), and attenuated NF-κB activation in astrocytes (König et al., 2014). These findings are of critical importance as elevated levels of Bid are seen both in the spinal cord and activated astrocytes in the SOD1^G93A^ transgenic mouse model (Guégan et al., 2002; König et al., 2014), suggesting increased Bid levels may contribute to the chronic inflammation evident in ALS disease progression. Interestingly, TNFα levels increase concurrent with disease progression in mutant SOD1-linked ALS pathogenesis (Hensley et al., 2003), and here we found reduced levels of TNFα in LPS-induced *bid*-deficient microglia. We have demonstrated that Bid is highly expressed in unstimulated microglia, and that microglial *bid* depletion attenuates SOD1^G93A^-induced toxicity. The role of microglia in SOD1-mediated toxicity has been supported by studies which demonstrate that accumulation of mutant SOD1 in either motoneurons alone (Pramatarova et al., 2001) or astrocytes (Gong et al., 2000) may not be sufficient to efficiently cause motoneuron degeneration, with the addition of SOD1^G93A^ microglia to co-cultures shown to induce motoneuron death (Zhao et al., 2010). Additionally, a recent study shows that NF-κB is activated concurrently with disease progression in SOD1^G93A^ mice, and that inhibition of NF-κB in microglia rescued motoneurons from cell death in SOD1^G93A^ murine cells in culture, and delayed motor function deficits in SOD1^G93A^ mice (Frakes et al., 2014).

The therapeutic potential of attenuated TLR4 signalling in ALS is highlighted in studies demonstrating reduced motoneuron degeneration in SOD1^G93A^ mice lacking *tlr4* (Lee et al., 2015), and in rescued SOD1^G93A^ stimulated motoneuron death *in vitro* using TLR4 inhibitors (De Paola et al., 2016). We demonstrate that Bid has a specific role in TLR4 signaling, as LPS primarily elicits its response via TLR4 and is a potent agonist of TLR4-mediated NF-κB activation (Hoshino et al., 1999; Bottcher et al., 2003), inducing the pro-inflammatory M1 activated microglial phenotype (Chhor et al., 2013). Acute LPS stimulations reveal decreased phosphorylation of IKKα and IKKβ, required for activation of the IKK complex and further signaling to IκBα (Wang et al., 2001). IκBα sequesters NF-κB subunits in the cytosol inhibiting their nuclear translocation, and *bid*-deficient microglia showed protracted IκBα degradation kinetics, indicating delayed TLR4 signaling (Mayo et al., 2011). Our data demonstrates decreased NF-κB activation levels in Bid-inhibited LPS-stimulated BV-2 cells. Constitutive activation of NF-κB in WT microglia induces both gliosis and motoneuron cell death (Frakes et al., 2014), and therefore ablating this activation may ameliorate both of these hallmarks of ALS aetiology, further highlighting a potential neuroprotective role for *bid* depletion in microglia.

TLR-NF-κB signaling is tightly regulated by a number of E3 ubiquitin ligase proteins. It has previously been reported that the E3 ubiquitin ligase Peli1 regulates the NF-κB pathway and reduced Peli1 levels are shown to impair TLR signaling (Jiang et al., 2003). Here we identified decreased levels of Peli1 upon acute LPS stimulation in *bid^−/−^* microglia compared with wild-type. As Peli1 facilitates the K63-linked ubiquitination of the IRAK complex (Butler et al., 2007; Ordureau et al., 2008), and subsequent downstream signaling to NF-κB, reduced Peli1 levels in *bid*-deficient microglia may suggest accelerated Peli1 degradation. We propose this to be a process by which Bid regulates TLR-induced NF-κB activation.

Many studies to date have shown that the TRAF6 RING domain is essential for the activation of IKK in TLR signaling by either activation of TAK1 (Lamothe et al., 2007; Walsh et al., 2008) or the Peli1-IRAK complex (Conze et al., 2008). Additionally, inhibiting the formation of Peli1-IRAK-TRAF6 interactions was identified to prevent the degradation of the NF-κB inhibitor IκBα (Choi et al., 2006). We propose that an interaction between Bid and the E3 ubiquitin ligase protein TRAF6 modulates the NF-κΒ signaling pathway in microglia. Together with previous studies, our data implies a potential cell-type-specific interaction between Bid and different signaling proteins essential for NF-κB activation. Interestingly the Bid-TRAF6 interaction we observe in microglia suggests that Bid may interact specifically with E3 ubiquitin ligase proteins, which regulate NF-κB activation. Ubiquitination of multiple proteins, forming mainly K63 and M1 chains (for review, see Iwai et al., 2014), is required for NF-κB activation, in order for the IKK complex to become phosphorylated with subsequent K48-linked proteasomal degradation of IκBα, resulting in the release NF-κB into the cytosol. TRAF6 activates NF-κB via facilitating the recruitment of the TAK1/TAB1/TAB2 complex (Lamothe et al., 2007) and by directly forming ubiquitin chains with NEMO (Gautheron et al., 2010) and IRAK-1 (Qian et al., 2001). We have demonstrated that Bid promotes the autoubiquitination of TRAF6, specifically K63-linked ubiquitination upon LPS stimulation. TRAF6 is autoubiquitinated by K63-linked chains, preventing the K48-linked degradation of TRAF6, and providing docking sites for an array of proteins that mediate NF-κB activation (Chen, 2005). The requirement of TRAF6 autoubiquitination for TRAF6-mediated ubiquitination of target proteins is debatable, with some studies arguing TRAF6 autoubiquitination to be unnecessary (Walsh et al., 2008; Wang et al., 2010) and other studies demonstrating that TRAF6 autoubiquitination is essential for TRAF6-mediated NEMO K63-ubiquitination and downstream NF-κB activation (Lamothe et al., 2007). Interestingly, it has been reported that unanchored polyubiquitin chains generated by TRAF6 can activate both TAK1 and IKK complexes (Xia et al., 2009).

Additionally, TLR4 is of increasing interest in ALS therapeutics, with another agonist of TLR4, high-mobility group box 1 (HMGB1), shown to be secreted from motoneurons and to have increased reactivity in astrocytes and microglia concurrent with ALS disease progression (Lo Coco et al., 2007; Lee et al., 2015). Of note, TNFα secretion from NF-κB-activated glial cells provides a death receptor agonist which may contribute to the rate of motoneuron death (Aebischer et al., 2013). We propose that the Bid-potentiated polyubiquitination of TRAF6 positively regulates TLR4-induced NF-κB signaling by promoting Peli1-IRAK-TRAF6 interactions and supporting proinflammatory signal propagation to IκBα and NF-κB. Furthermore, as NF-κB is a complex therapeutic target, a cell-specific approach may prove more effective. Depletion of microglial Bid offers a potential avenue for the regulation and attenuation of the TLR4- and TRAF6-mediated inflammatory response in ALS pathogenesis.

## Synthesis

The decision was a result of the Reviewing Editor Jorg Schulz and the peer reviewers coming together and discussing their recommendations until a consensus was reached. A fact-based synthesis statement explaining their decision and outlining what is needed to prepare a revision is listed below. The following reviewers agreed to reveal their identity: Xugang Xia, Melissa Bowerman

While the revisions were extensive and improved the manuscript, many important issues still remain. In a revision, please pay more attention to the concepts on ALS pathobiology as the description in the Introduction and Discussion contradicts with commonly accepted knowledge. Microglia is commonly activated in response to neuronal damage in animal models and human patients with neurological diseases including ALS. The differential effect on TLR2 and TLR4 between WT and mutant SOD1 is not convincing. How the conditioned medium from sick neurons stimulates microglia to become active is not known and how this event is related to TLR2 and TLR4 activity in microglia is not clear. Apparently, these studies are dispensable for the conclusion, but these weaknesses dampen the enthusiasm for this study. Unfortunately, the authors did not pay attention to the comments on this issue raised in the previous review. If authors insist on including these data, they may wish to discuss the relevance of the studies to the conclusion in Discussion.

Furthermore, please address in a revision the following issues:

• Figures 1 B and D still do not appear to show increased tlr4 expression, which is the foundation of the manuscript and ensuing experiments.

• Figures 1E and F: one-tailed t-test used instead of two-tailed. Why? This would allow to reach statistical significance as it only takes into account the effect in one direction.

• Figure 3C, better to place the wt and bid-/- data in an interleaved fashion per time-point to better visualize and quantify the reduced effect of LPS on pIKKa/b ratios

• Figure 3G, place data in interleaved fashion. It is unclear if significant difference is between wt and bid-/- at 1 hour.

• Figure 3J: data does not appear significantly different. The authors present the data as expression ratios and discuss the data in the text as fold differences.

• Figure 4B: again, interleaved data presentation would make for a stronger visual point.

• Figure 5A: immunoblots are of very poor quality and unconvincing. Especially as they have been performed in separate experiments (as indicated by separation), one could question if different exposure times were used.

• Figure 5B: Again, very poor quality and unconvincing and the immunoblot has been extensively cropped, leaving us to think that bands may be present in the lower part in the IgG IP area.

• Figure 5C: where is the representative example of vehicle condition (no LPS)

• Figure 5D: again, the authors discuss the fold differences without it being clear if there is a significant increase in the bid/traf6 interaction in LPS-treated cells.

• Page 19, line 427, data obtained in Figure 6A are not discussed.

• Figures 6B, C, D: no significant differences but data is discussed as such.

• Figure 6F: Unclear what the linked dots are supposed to represent.
